# A Survey of Robotic Harvesting Systems and Enabling Technologies

**DOI:** 10.1007/s10846-022-01793-z

**Published:** 2023-01-27

**Authors:** Leonidas Droukas, Zoe Doulgeri, Nikolaos L. Tsakiridis, Dimitra Triantafyllou, Ioannis Kleitsiotis, Ioannis Mariolis, Dimitrios Giakoumis, Dimitrios Tzovaras, Dimitrios Kateris, Dionysis Bochtis

**Affiliations:** 1 Department of Electrical and Computer Engineering, Aristotle University of Thessaloniki (AUTH), Thessaloniki, 54124 Greece; 2 Information Technologies Institute (ITI), Centre for Research and Technology Hellas (CERTH), Thessaloniki, 57001 Greece; 3 Institute for Bio-Economy and Agri-Technology (iBO), Centre for Research and Technology Hellas (CERTH), Volos, 38333 Greece

**Keywords:** Robotic harvesting, Automated agriculture, Agricultural functionalities, State-of-art review

## Abstract

This paper presents a comprehensive review of ground agricultural robotic systems and applications with special focus on harvesting that span research and commercial products and results, as well as their enabling technologies. The majority of literature concerns the development of crop detection, field navigation via vision and their related challenges. Health monitoring, yield estimation, water status inspection, seed planting and weed removal are frequently encountered tasks. Regarding robotic harvesting, apples, strawberries, tomatoes and sweet peppers are mainly the crops considered in publications, research projects and commercial products. The reported harvesting agricultural robotic solutions, typically consist of a mobile platform, a single robotic arm/manipulator and various navigation/vision systems. This paper reviews reported development of specific functionalities and hardware, typically required by an operating agricultural robot harvester; they include (a) vision systems, (b) motion planning/navigation methodologies (for the robotic platform and/or arm), (c) Human-Robot-Interaction (HRI) strategies with 3D visualization, (d) system operation planning & grasping strategies and (e) robotic end-effector/gripper design. Clearly, automated agriculture and specifically autonomous harvesting via robotic systems is a research area that remains wide open, offering several challenges where new contributions can be made.

## References

[1] Eberhardt M, Vollrath D (2018). The effect of agricultural technology on the speed of development. World Dev..

[2] Fathallah FA (2010). Musculoskeletal disorders in labor-intensive agriculture. Appl. Ergon..

[3] Proto AR, Zimbalatti G (2010). Risk assessment of repetitive movements in the citrus fruit industry. J. Agric. Saf. Health.

[4] Zhang Z, Wang Y, Zhang Z, Li D, Wu Z, Bai R, Meng G (2019). Ergonomic and efficiency analysis of conventional apple harvest process. Int. J. Agric. Biol. Eng..

[5] Zhang Z, Zhang Z, Wang X, Liu H, Wang Y, Wang W (2019). Models for economic evaluation of multi-purpose apple harvest platform and software development. Int. J. Agric. Biol. Eng..

[6] Marinoudi V, Sørensen CG, Pearson S, Bochtis D (2019). Robotics and labour in agriculture. a context consideration. Biosyst. Eng..

[7] Fountas S, Mylonas N, Malounas I, Rodias E, Santos CH, Pekkeriet E (2020). Agricultural robotics for field operations. Sensors.

[8] Shamshiri RR, Weltzien C, Hameed IA, Yule IJ, Grift TE, Balasundram SK, Pitonakova L, Ahmad D, Chowdhary G (2018). Research and development in agricultural robotics: a perspective of digital farming. Int. J. Agric. Biol. Eng..

[9] Bac CW, van Henten EJ, Hemming J, Edan Y (2014). Harvesting robots for high-value crops: State-of-the-art review and challenges ahead. J. Field Robot..

[10] Oliveira, L.F.P., Moreira, A.P., Silva, M.F.: Advances in agriculture robotics: A state-of-the-art review and challenges ahead. Robotics 10(2). 10.3390/robotics10020052 (2021)

[11] Slaughter DC, Giles DK, Downey D (2008). Autonomous robotic weed control systems: a review. Comput. Electron. Agric..

[12] Zhang, Q., Karkee, M., Tabb, A.: The use of agricultural robots in orchard management. Burleigh Dodds Series in Agricultural Science, 187–214. 10.19103/as.2019.0056.14 (2019)

[13] Bechar, A., Vigneault, C.: Agricultural robots for field operations. Part 2: Operat. Syst. Biosyst. Eng., 110–128. 10.1016/j.biosystemseng.2016.11.004 (2017)

[14] Saiz-Rubio, V., Rovira-Más, F.: From smart farming towards agriculture 5.0: a review on crop data management. Agronomy (2)207. 10.3390/agronomy10020207 (2020)

[15] Baillie, C.P., Thomasson, J.A., Lobsey, C.R., McCarthy, C.L., Antille, D.L.: A review of the state of the art in agricultural automation part i: Sensing technologies for optimization of machine operation and farm inputs. 10.13031/aim.201801589 (2018)

[16] Fue, K.G., Porter, W.M., Barnes, E.M., Rains, G.C.: An extensive review of mobile agricultural robotics for field operations: Focus on cotton harvesting. AgriEngineering (1)150–174. 10.3390/agriengineering2010010 (2020)

[17] Defterli, S.G., Shi, Y., Xu, Y., Ehsani, R.: Review of robotic technology for strawberry production. Appl. Eng. Agric. (3)301–318. 10.13031/aea.32.11318 (2016)

[18] Aravind, K.R., Raja, P., Pérez-Ruiz, M.: Task-based agricultural mobile robots in arable farming: a review. Spanish Journal of Agricultural Research (1)16. 10.5424/sjar/2017151-9573 (2017)

[19] Tsolakis, N., Bechtsis, D., Bochtis, D.: Agros: a robot operating system based emulation tool for agricultural robotics. Agronomy (7)403. 10.3390/agronomy9070403 (2019)

[20] del Cerro, J., Cruz Ulloa, C., Barrientos, A., de León Rivas, J.: Unmanned aerial vehicles in agriculture: A survey. Agronomy 11(2). 10.3390/agronomy11020203 (2021)

[21] Mogili UR, Deepak BBVL (2018). Review on application of drone systems in precision agriculture. Procedia Comput. Sci..

[22] Kim J, Kim S, Ju C, Son HI (2019). Unmanned aerial vehicles in agriculture: A review of perspective of platform, control, and applications. IEEE Access.

[23] Feng L, Chen S, Zhang C, Zhang Y, He Y (2021). A comprehensive review on recent applications of unmanned aerial vehicle remote sensing with various sensors for high-throughput plant phenotyping. Comput. Electron. Agric..

[24] Ju C, Kim J, Seol J, Son HI (2022). A review on multirobot systems in agriculture. Comput. Electron. Agric..

[25] Ribeiro A, Conesa-Muñoz J (2021). Multi-robot Systems for Precision Agriculture.

[26] Bhandari, S., Raheja, A., Green, R.L., Do, D.: Towards collaboration between unmanned aerial and ground vehicles for precision agriculture. In: Proc. SPIE, Autonomous Air and Ground Sensing Systems for Agricultural Optimization and Phenotyping II, vol. 10218, p. 1021806. 10.1117/12.2262049 (2017)

[27] Pretto A, Aravecchia S, Burgard W, Chebrolu N, Dornhege C, Falck T, Fleckenstein F, Fontenla A, Imperoli M, Khanna R, Liebisch F, Lottes P, Milioto A, Nardi D, Nardi S, Pfeifer J, Popović M, Potena C, Pradalier C, Rothacker-Feder E, Sa I, Schaefer A, Siegwart R, Stachniss C, Walter A, Winterhalter W, Wu X, Nieto J (2021). Building an aerial–ground robotics system for precision farming: an adaptable solution. IEEE Robot Autom Mag.

[28] Deusdado, P., Pinto, E., Guedes, M., Marques, F., Rodrigues, P., Lourenço, A., Mendonça, R., Silva, A., Santana, P., Corisco, J., Almeida, M., Portugal, L., Caldeira, R., Barata, J., Flores, L.: An aerial-ground robotic team for systematic soil and biota sampling in estuarine mudflats. In: Robot 2015: Second Iberian robotics conference, pp. 15–26. Springer. 10.1007/978-3-319-27149-1_2 (2016)

[29] Edmonds, M., Yigit, T., Yi, J.: Resolution-optimal, energy-constrained mission planning for unmanned aerial/ground crop inspections. In: 2021 IEEE 17Th International conference on automation science and engineering (CASE), pp. 2235–2240. 10.1109/CASE49439.2021.9551394 (2021)

[30] Li, P., Lee, S., Hsu, H.-Y.: Review on fruit harvesting method for potential use of automatic fruit harvesting systems. Procedia Eng, 351–366. 10.1016/j.proeng.2011.11.2514 (2011)

[31] Coppock, G.E., Hedden, S.L.: Design and development of a tree-shaker harvest system for citrus fruit. Transactions of the ASAE (3)339–342. 10.13031/2013.39404 (1968)

[32] Sumner, H.R., Hedden, S.L., Alfred, L.: Harvesting oranges with a full-powered positiong limb shaker. Floriad State Horticultural Society, 117–120 (1975)

[33] Torregrosa A, Ortí E, Martín B, Gil J, Ortiz C (2009). Mechanical harvesting of oranges and mandarins in spain. Biosyst. Eng..

[34] Silwal A, Davidson JR, Karkee M, Mo C, Zhang Q, Lewis K (2017). Design, integration, and field evaluation of a robotic apple harvester. J Field Robot.

[35] Baeten J, Donné K, Boedrij S, Beckers W, Claesen E (2008). Autonomous fruit picking machine: a robotic apple harvester. Field and Service Robotics.

[36] Bulanon DM, Kataoka T (2010). A fruit detection system and an end effector for robotic harvesting of fuji apples. Agric. Eng. Int.: CIGR J..

[37] Onishi Y, Yoshida T, Kurita H, Fukao T, Arihara H, Iwai A (2019). An automated fruit harvesting robot by using deep learning. ROBOMECH J..

[38] De-An Z, Jidong L, Wei J, Ying Z, Yu C (2011). Design and control of an apple harvesting robot. Biosyst. Eng..

[39] Davidson, J.R., Hohimer, C.J., Mo, C., Karkee, M.: Dual robot coordination for apple harvesting. **110**(2)112–122. 10.13031/aim.201700567 (2017)

[40] Muscato G, Prestifilippo M, Abbate N, Rizzuto I (2005). A prototype of an orange picking robot: past history, the new robot and experimental results. Ind. Robot..

[41] Klaoudatos, D.S., Moulianitis, V.C., Aspragathos, N.A.: Development of an experimental strawberry harvesting robotic system. **2**,437–445. 10.5220/0007934004370445 (2019)

[42] Xiong Y, Peng C, Grimstad L, From PJ, Isler V (2019). Development and field evaluation of a strawberry harvesting robot with a cable-driven gripper. Comput. Electron. Agric..

[43] Xiong Y, Ge Y, Grimstad L, From PJ (2020). An autonomous strawberry-harvesting robot: design, development, integration, and field evaluation. J. Field Robot..

[44] Hayashi S, Yamamoto S, Tsubota S, Ochiai Y, Kobayashi K, Kamata J, Kurita M, Kurita M, Inazumi H, Rajendra P (2014). Automation technologies for strawberry harvesting and packing operations in japan. J. Berry Res..

[45] Hayashi S, Shigematsu K, Yamamoto S, Kobayashi K, Kohno Y, Kamata J, Kurita M (2010). Evaluation of a strawberry-harvesting robot in a field test. Biosyst. Eng..

[46] Tanigaki K, Fujiura T, Akase A, Imagawa J (2008). Cherry-harvesting robot. Comput. Electron. Agric..

[47] Edan Y, Rogozin D, Flash T, Miles GE (2000). Robotic melon harvesting. IEEE Trans. Robot. Autom..

[48] Bechar A, Edan Y (2003). Human-robot collaboration for improved target recognition of agricultural robots. Ind. Robot..

[49] Umeda M, Kubota S, Iida M (1999). Development of ’stork’, a watermelon-harvesting robot. Artif. Life Robot..

[50] Zion B, Mann M, Levin D, Shilo A, Rubinstein D, Shmulevich I (2014). Harvest-order planning for a multiarm robotic harvester. Comput. Electron. Agric..

[51] Ceres R, Pons JL, Jimenez AR, Martin JM, Calderon L (1998). Design and implementation of an aided fruit-harvesting robot(agribot). Ind. Robot..

[52] Yaguchi, H., Nagahama, K., Hasegawa, T., Inaba, M.: Development of an autonomous tomato harvesting robot with rotational plucking gripper. **3**, 652–657. 10.1109/IROS.2016.7759122 (2016)

[53] Wang LL, Bo Z, Jinwei F, Xiaoan H, Shu W, Yashuo L, Zhou Q, Chongfeng W (2017). Development of a tomato harvesting robot used in greenhouse. Int. J. Agric. Biol. Eng..

[54] Feng Q, Zou W, Fan P, Zhang C, Wang X (2018). Design and test of robotic harvesting system for cherry tomato. Int. J. Agric. Biol. Eng..

[55] Zhao Y, Gong L, Liu C, Huang Y (2016). Dual-arm robot design and testing for harvesting tomato in greenhouse. IFAC-PapersOnLine.

[56] Ling X, Zhao Y, Gong L, Liu C, Wang T (2019). Dual-arm cooperation and implementing for robotic harvesting tomato using binocular vision. Robot. Auton. Syst..

[57] Henten EJV, Hemming J, van Tuijl BAJ, Kornet JG, Meuleman J, Bontsema J, van Os EA (2002). An autonomous robot for harvesting cucumbers in greenhouses. Auton. Robot..

[58] Herck, L.V., Kurtser, P., Wittemans, L., Edan, Y.: Crop design for improved robotic harvesting: a case study of sweet pepper harvesting. Biosyst. Eng. **192**, 294–308. 10.1016/j.biosystemseng.2020.01.021 (2020)

[59] Bloch V, Degani A, Bechar A (2018). A methodology of orchard architecture design for an optimal harvesting robot. Biosyst. Eng..

[60] Hayashi S, Ganno K, Ishii Y, Tanaka I (2002). Robotic harvesting system for eggplants. Japan Agricultural Research Quarterly: JARQ.

[61] Foglia MM, Reina G (2006). Agricultural robot for radicchio harvesting. J. Field Robot..

[62] Irie, N., Taguchi, N., Horie, T., Ishimatsu, T.: Asparagus harvesting robot coordinated with 3-d vision sensor, 1–6. 10.1109/ICIT.2009.4939556 (2009)

[63] Zhang T, Huang Z, You W, Lin J, Tang X, Huang H (2020). An autonomous fruit and vegetable harvester with a low-cost gripper using a 3d sensor. Sensors.

[64] Kitamura, S., Oka, K.: Recognition and cutting system of sweet pepper for picking robot in greenhouse horticulture 1807–1812. 10.1109/icma.2005.1626834 (2005)

[65] Lehnert C, English A, McCool C, Tow AW, Perez T (2017). Autonomous sweet pepper harvesting for protected cropping systems. IEEE Robotics and Automation Letters.

[66] Arad B, Balendonck J, Barth R, Ben-Shahar O, Edan Y, Hellström T, Hemming J, Kurtser P, Ringdahl O, Tielen T, van Tuijl B (2020). Development of a sweet pepper harvesting robot. Journal of Field Robotics.

[67] Quaglia G, Visconte C, Scimmi LS, Melchiorre M, Cavallone P, Pastorelli S (2020). Design of a ugv powered by solar energy for precision agriculture. Robotics.

[68] Quaglia G, Cavallone P, Visconte C (2019). Agri-q: Agriculture ugv for monitoring and drone landing. IFToMM Symposium on Mechanism Design for Robotics.

[69] Quaglia G, Visconte C, Scimmi LS, Melchiorre M, Cavallone P, Pastorelli S (2019). Design of the positioning mechanism of an unmanned ground vehicle for precision agriculture. IFToMM World Congress on Mechanism and Machine Science.

[70] Monta, M., Kondo, N., Shibano, Y.: Agricultural robot in grape production system. **3**, 2504–2509. 10.1109/ROBOT.1995.525635 (1995)

[71] Ogawa Y, Kondo NA, Monta MI, Sakaeshibusawa N (2006). Spraying robot for grape production. Field and Service Robotics.

[72] Oberti R, Marchi M, Tirelli P, Calcante A, Iriti M, Hočevar M, Baur J, Pfaff J, Schütz C, Ulbrich H (2013). Selective spraying of grapevine’s diseases by a modular agricultural robot. J. Agric. Eng..

[73] Bontsema, J., Hemming, J., Pekkeriet, E., Saeys, W., Edan, Y., Shapiro, A., Hocevar, M., Oberti, R., Armada, M., Ulbrich, H., Baur, J., Debilde, B., Best, S., Evain, S., Gauchel, W., Hellström, T., Ringdahl, O.: Crops: Clever robots for crops. Engineering & Technology Reference, 1–11. 10.1049/etr.2015.0015 (2015)

[74] Roure, F., Moreno, G., Soler, M., Faconti, D., Serrano, D., Astolfi, P., Bardaro, G., Gabrielli, A., Bascetta, L., Matteucci, M.: Grape: Ground robot for vineyard monitoring and protection. 249–260, 10.1007/978-3-319-70833-1_21 (2017)

[75] Reiser D, Sehsah ES, Bumann O, Morhard J, Griepentrog HW (2019). Development of an autonomous electric robot implement for intra-row weeding in vineyards. Agriculture.

[76] Santos, F.N.D., Sobreira, H.M., Campos, D.F., Morais, R., Moreira, A.P., Contente, O.: Towards a reliable monitoring robot for mountain vineyards. 37–43, 10.11009/2015.21 (2015)

[77] Botterill T, Paulin S, Green R, Williams S, Lin J, Saxton V, Mills S, Chen X, Corbett-Davies S (2017). A robot system for pruning grape vines. J. Field Robot..

[78] Tang, Y., Chen, M., Wang, C., Luo, L., Li, J., Lian, G., Zou, X.: Recognition and localization methods for vision-based fruit picking robots: A review. Frontiers in Plant Science 11. 10.3389/fpls.2020.00510(2020) 10.3389/fpls.2020.00510 PMC7250149 32508853

[79] Saleem MH, Potgieter J, Arif KM (2021). Automation in agriculture by machine and deep learning techniques: a review of recent developments. Precision Agric..

[80] Vitzrabin E, Edan Y (2016). Adaptive thresholding with fusion using a rgbd sensor for red sweet-pepper detection. Biosys. Eng..

[81] Blok PM, Barth R, Van den Berg W (2016). Machine vision for a selective broccoli harvesting robot. IFAC-PapersOnLine.

[82] Kurtser P, Ringdahl O, Rotstein N, Berenstein R, Edan Y (2020). In-field grape cluster size assessment for vine yield estimation using a mobile robot and a consumer level rgb-d camera. IEEE Robot. Automation Lett..

[83] Nuske S, Wilshusen K, Achar S, Yoder L, Narasimhan S, Singh S (2014). Automated visual yield estimation in vineyards. J. Field Robot..

[84] Luo L, Tang Y, Lu Q, Chen X, Zhang P, Zou X (2018). A vision methodology for harvesting robot to detect cutting points on peduncles of double overlapping grape clusters in a vineyard. Comput. Industry.

[85] Ostovar A, Ringdahl O, Hellstrom T (2018). Adaptive image thresholding of yellow peppers for a harvesting robot. Robotics.

[86] Altaheri H, Alsulaiman M, Muhammad G (2019). Date fruit classification for robotic harvesting in a natural environment using deep learning. IEEE Access.

[87] Ge Y, Xiong Y, Tenorio GL, From PJ (2019). Fruit localization and environment perception for strawberry harvesting robots. IEEE Access.

[88] Lehnert, C., Sa, I., McCool, C., Upcroft, B., Perez, T.: Sweet pepper pose detection and grasping for automated crop harvesting, 2428–2434. 10.1109/ICRA.2016.7487394 (2016)

[89] Kang, H., Zhou, H., Chen, C.: Visual perception and modeling for autonomous apple harvesting. IEEE Access, 62151–62163. 10.1109/ACCESS.2020.2984556 (2020)

[90] Zapotezny-Anderson PM, Lehnert C (2019). Towards active robotic vision in agriculture: a deep learning approach to visual servoing in occluded and unstructured protected cropping environments. IFAC-PapersOnLine.

[91] Arad B, Kurtser P, Barnea E, Harel B, Edan Y, Ben-Shahar O (2019). Controlled lighting and illumination-independent target detection for real-time cost-efficient applications. the case study of sweet pepper robotic harvesting. Sensors.

[92] Tian Y, Duan H, Luo R, Zhang Y, Jia W, Lian J, Zheng Y, Ruan C, Li C (2019). Fast recognition and location of target fruit based on depth information. IEEE Access.

[93] Kurtser P, Ringdahl O, Rotstein N, Andreasson H (2020). Pointnet and geometric reasoning for detection of grape vines from single frame rgb-d data in outdoor conditions. Proceedings of the Northern Lights Deep Learning Workshop 2020.

[94] McCool, C., Sa, I., Dayoub, F., Lehnert, C., Perez, T., Upcroft, B.: Visual detection of occluded crop: For automated harvesting. 2506–2512. 10.1109/ICRA.2016.7487405 (2016)

[95] Liu, Y.P., Yang, C.H., Ling, H., Mabu, S., Kuremoto, T.: A visual system of citrus picking robot using convolutional neural networks. 344–349. 10.1109/ICSAI.2018.8599325 (2018)

[96] Lehnert, C., Tsai, D., Eriksson, A., McCool, C.: 3d move to see: Multiperspective visual servoing towards the next best view within unstructured and occluded environments. 3890–3897. 10.1109/IROS40897.2019.8967918 (2019)

[97] Zemmour E, Kurtser P, Edan Y (2019). Automatic parameter tuning for adaptive thresholding in fruit detection. Sensors.

[98] Vitzrabin E, Edan Y (2016). Changing task objectives for improved sweet pepper detection for robotic harvesting. IEEE Robot. Automat. Lett..

[99] Pothen, Z.S., Nuske, S.: Texture-based fruit detection via images using the smooth patterns on the fruit. 5171–5176. 10.1109/ICRA.2016.7487722 (2016)

[100] Bargoti, S., Underwood, J.P.: Image segmentation for fruit detection and yield estimation in apple orchards. Journal of Field Robotics **34**(6) (2016). 10.1002/rob.21699

[101] Lin G, Tang Y-C, Zou X, Xiong J, Li J (2019). Guava detection and pose estimation using a low-cost rgb-d sensor in the field. Sensors.

[102] Fawakherji, M., Youssef, A., Bloisi, D., Pretto, A., Nardi, D.: Crop and weeds classification for precision agriculture using context-independent pixel-wise segmentation, 146–152. 10.1109/IRC.2019.00029(2019)

[103] Häni N, Roy P, Isler V (2020). A comparative study of fruit detection and counting methods for yield mapping in apple orchards. J. Field Robot..

[104] Liu, X., Chen, S., Aditya, S., Sivakumar, N., Dcunha, S., Qu, C., Taylor, C.J., Das, J., Kumar, V.: Robust fruit counting: Combining deep learning, tracking, and structure from motion, 1045–1052. 10.1109/IROS.2018.8594239 (2018)

[105] Lin G, Tang Y, Zou X, Xiong J, Fang Y (2019). Color-, depth-, and shape-based 3d fruit detection. Precis. Agric..

[106] McCool C, Perez T, Upcroft B (2017). Mixtures of lightweight deep convolutional neural networks: Applied to agricultural robotics. IEEE Robot. Autom. Lett..

[107] Santos, T.T., Souza, L., dos Santos, A.A., Avila, S.: Grape detection, segmentation and tracking using deep neural networks and three dimensional association. Comput. Electron. Agric., 170. 10.1016/j.compag.2020.105247 (2020)

[108] Koirala A, Walsh K, Wang Z, McCarthy C (2019). Deep learning for realtime fruit detection and orchard fruit load estimation: benchmarking of mangoyolo. Precis. Agric..

[109] Tian Y, Yang G, Wang Z, Li E, Liang Z (2019). Detection of apple lesions in orchards based on deep learning methods of cyclegan and yolov3-dense. Journal of Sensors.

[110] Gonzalez S, Arellano C, Tapia JE (2019). Deepblueberry: Quantification of blueberries in the wild using instance segmentation. IEEE Access.

[111] Ganesh P, Volle K, Burks T, Mehta S (2019). Deep orange: Mask r-cnn based orange detection and segmentation. IFAC-PapersOnLine.

[112] Sa, I., Ge, Z., Dayoub, F., Upcroft, B., Perez, T., McCool, C.: Deepfruits: A fruit detection system using deep neural networks. Sensors (Basel Switzerland) 16(8). 10.3390/s16081222 (2016) 10.3390/s16081222 PMC5017387 27527168

[113] Gene-Mola J, Vilaplana V, Rosell-Polo JR, Morros JR, Ruiz-Hidalgo J, Gregorio E (2019). Multi-modal deep learning for fuji apple detection using rgb-d cameras and their radiometric capabilities. Comput. Electron. Agric..

[114] Hasan, M.M., Chopin, J.P., Laga, H., Miklavcic, S.J.: Detection and analysis of wheat spikes using convolutional neural networks. Plant Methods 14(100). 10.1186/s13007-018-0366-8 (2018) 10.1186/s13007-018-0366-8 PMC6236889 30459822

[115] Kang, H., Chen, C.: Fast implementation of real-time fruit detection in apple orchards using deep learning. Comput. Electron. Agric., 168. 10.1016/j.compag.2019.105108 (2020)

[116] Kang, H., Chen, C.: Fruit detection and segmentation for apple harvesting using visual sensor in orchards. Sensors (Basel Switzerland) 19(20). 10.3390/s19204599 (2019) 10.3390/s19204599 PMC6832306 31652634

[117] Kirk R, Cielniak G, Mangan M (2020). L*a*b*fruits: a rapid and robust outdoor fruit detection system combining bio-inspired features with one-stage deep learning networks. Sensors.

[118] Dias PA, Tabb A, Medeiros H (2018). Apple flower detection using deep convolutional networks. Comput. Ind..

[119] Bayati, M., Fotouhi, R.: A mobile robotic platform for crop monitoring. Advances in Robotics and Automation 7(1). 10.4172/2168-9695.1000186 (2018)

[120] Virlet N, Sabermanesh K, Sadeghi-Tehran P, Hawkesford MJ (2017). Field scanalyzer: an automated robotic field phenotyping platform for detailed crop monitoring. Funct. Plant Biol..

[121] Shafiekhani A, Kadam S, Fritschi FB, DeSouza GN (2017). Vinobot and vinoculer: Two robotic platforms for high-throughput field phenotyping. Sensors.

[122] Gutiérrez S, Fernández-Novales J, Diago MP, Tardaguila J (2018). On-the-go hyperspectral imaging under field conditions and machine learning for the classification of grapevine varieties. Frontiers in Plant Sci..

[123] Hu K, Coleman GRY, Zeng S, Wang Z, Walsh M (2020). Graph weeds net: a graph-based deep learning method for weed recognition. Comput. Electron. Agric..

[124] Christiansen P, Nielsen L, Steen K, Jorgensen R, Karstoft H (2016). Deepanomaly: Combining background subtraction and deep learning for detecting obstacles and anomalies in an agricultural field. Sensors.

[125] Isokane, T., Okura, F., Ide, A., Matsushita, Y., Yagi, Y.: Probabilistic plant modeling via multi-view image-to-image translation. 2906–2915, 10.1109/CVPR.2018.00307 (2018)

[126] Bietresato M, Carabin G, Vidoni R, Gasparetto A, Mazzetto F (2016). Evaluation of a lidar-based 3d-stereoscopic vision system for crop-monitoring applications. Comput. Electron. Agric..

[127] Williams D, Britten A, McCallum S, Jones H, Aitkenhead M, Karley A, Loades K, Prashar A, Graham J (2017). A method for automatic segmentation and splitting of hyperspectral images of raspberry plants collected in field conditions. Plant methods.

[128] Daniels AJ, Poblete-Echeverría C, Opara UL, Nieuwoudt HH (2019). Measuring internal maturity parameters contactless on intact table grape bunches using nir spectroscopy. Front. Plant Sci..

[129] Costa DDS, Mesa NFO, Freire MS, Ramos RP, Mederos BJT (2019). Development of predictive models for quality and maturation stage attributes of wine grapes using vis-nir reflectance spectroscopy. Postharvest Biology and Technol..

[130] Porep, J.U., Mattes, A., Nikfardjam, M.S., Kammerer, D.R., Carle, R.: Implementation of an on-line near infrared/visible (nir/vis) spectrometer for rapid quality assessment of grapes upon receival at wineries. Australian Journal of Grape and Wine Research 21(1). 10.1111/ajgw.12120 (2015)

[131] Giovenzana V, Beghi R, Malegori C, Civelli R, Guidetti R (2013). Wavelength selection with a view to a simplified handheld optical system to estimate grape ripeness. Am. J. Enol. Vitic..

[132] Wendel A, Underwood J, Walsh K (2018). Maturity estimation of mangoes using hyperspectral imaging from a ground based mobile platform. Comput. Electron. Agric..

[133] Gutiérrez S, Wendel A, Underwood J (2019). Ground based hyperspectral imaging for extensive mango yield estimation. Comput. Electron. Agric..

[134] Gutiérrez S, Wendel A, Underwood J (2019). Spectral filter design based on in-field hyperspectral imaging and machine learning for mango ripeness estimation. Comput. Electron. Agric..

[135] Nasirahmadi, A., Wilczek, U., Hensel, O.: Sugar beet detection during harvesting using different convolutional neural networok models. Agriculture 11(11). 10.3390/agriculture11111111 (2021)

[136] Tian, S., Wang, S., Xu, H.: Early detection of freezing damage in oranges by online vis/nir transmission coupled with diameter method and deep 1d-cnn. Comput. Electron. Agric., 193. 10.1016/j.compag.2021.106638 (2022)

[137] Jin X, Jie L, Wang S, Qi HJ, Li SW (2018). Classifying wheat hyperspectral pixels of healthy heads and fusarium head blight disease using a deep neural network in the wild field. Remote Sens..

[138] Mack J, Lenz C, Teutrine J, Steinhage V (2017). High-precision 3d detection and reconstruction of grapes from laser range data for efficient phenotyping based on supervised learning. Comput. Electron. Agric..

[139] Hafeez, A., Aslam Husain, M., Singh, S.P., Chauhan, A., Khan, M.T., Kumar, N., Chauhan, A., Soni, S.K.: Implementation of drone technology for farm monitoring and pesticide spraying: A review. Information Processing in Agriculture. 10.1016/j.inpa.2022.02.002 (2022)

[140] Esposito, M., Crimaldi, V.M., et al.: Cirillo Drone and sensor technology for sustainable weed management: a review. Chem. Biol. Technol Agric **8**(18). 10.1186/s40538-021-00217-8 (2022)

[141] Su J (2021). Aerial visual perception in smart farming: Field study of wheat yellow rust monitoring. IEEE Trans Industr Inform.

[142] Crimaldi, M., Cristiano, V., De Vivo, A., Isernia, M., Ivanov, P., Sarghini, F.: Neural network algorithms for real time plant diseases detection using uavs. Innovative Biosystems Engineering for Sustainable Agriculture, 67. 10.1007/978-3-030-39299-4_89 (2020)

[143] Pflanz, M., Schirrmann, M., Nordmeyer, H.: Drone based weed monitoring with an image feature classifier. Julius-Kühn-Archiv, 84 (2018)

[144] Liao, J., Babiker, I., Xie, W.-F., Li, W., Cao, L.: Dandelion segmentation with background transfer learning and rgb-attention module. Comput. Electron. Agric., 202. 10.1016/j.compag.2022.107355(2022)

[145] Sa, I., Popović, M., Khanna, R., Chen, Z., Lottes, P., Liebisch, F., Nieto, J., Stachniss, C., Walter, A., Siegwart, R.: Weedmap: a large-scale semantic weed mapping framework using aerial multispectral imaging and deep neural network for precision farming. Remote Sensing 10(9). 10.3390/rs10091423(2018)

[146] Mazzia, V., Comba, L., Khaliq, A., Chiaberge, M., Gay, P.: Uav and machine learning based refinement of a satellite-driven vegetation index for precision agriculture. Sensors 20(9). 10.3390/s20092530 (2020) 10.3390/s20092530 PMC7249115 32365636

[147] Candiago S, Remondino F, De Giglio M, Dubbini M, Gattelli M (2015). Evaluating multispectral images and vegetation indices for precision farming applications from uav images. Remote Sens..

[148] Che’Ya, N.N., Dunwoody, E., Gupta, M.: Assessment of weed classification using hyperspectral reflectance and optimal multispectral uav imagery. Agronomy 11(7). 10.3390/agronomy11071435 (2021)

[149] Mink R, Linn AI, Santel H-J, Gerhards R (2019). Sensor-based evaluation of maize (zea mays) and weed response to post-emergence herbicide applications of isoxaflutole and cyprosulfamide applied as crop seed treatment or herbicide mixing partner. Pest Manag. Sci..

[150] Zaidner G, Shapiro A (2016). A novel data fusion algorithm for low-cost localisation and navigation of autonomous vineyard sprayer robots. Biosyst. Eng..

[151] Gan H, Lee WS (2018). Development of a navigation system for a smart farm. IFAC-PapersOnLine.

[152] Biber, P., Weiss, U., Dorna, M., Albert, A: Navigation system of the autonomous agricultural robot ‘bonirob,’. Workshop on Agricultural Robotics: Enabling Safe, Efficient, and Affordable Robots for Food Production (collocated with IROS 2012) Portugal (2012)

[153] Gu Y, Li Z, Zhang Z, Li J, Chen L (2020). Path tracking control of field information- collecting robot based on improved convolutional neural network algorithm. Sensors.

[154] Ouellette, R., Hirasawa, K.: Mayfly: a small mapping robot for japanese office environments. In: IEEE/ASME International Conference on Advanced Intelligent Mechatronics (AIM), 880–885. 10.1109/AIM.2008.4601777 (2008)

[155] Zhang, J., Maeta, S., Bergerman, M., Singh, S.: Mapping orchards for autonomous navigation. ASABE and CSBE/SCGAB Annual International Meeting, St. Joseph, Michigan, American Society of Agricultural and Biological Engineers. 10.13031/aim.20141838567 (2014)

[156] Libby, J., Kantor, G.: Deployment of a point and line feature localization system for an outdoor agriculture vehicle, pp 1565–1570. 10.1109/ICRA.2011.5980430 (2011)

[157] Jin J, Tang L (2009). Corn plant sensing using real-time stereo vision. J. Field Robot..

[158] Cadena C, Carlone L, Carrillo H, Latif Y, Scaramuzza D, Neira J, Reid I, Leonard JJ (2016). Past, present, and future of simultaneous localization and mapping: Toward the robust-perception age. IEEE Trans. Robot..

[159] Cheein FA, Steiner G, Paina GP, Carelli R (2011). Optimized eif-slam algorithm for precision agriculture mapping based on stems detection. Comput. Electron. Agric..

[160] Pierzchała M, Giguère P, Astrup R (2018). Mapping forests using an unmanned ground vehicle with 3d lidar and graph-slam. Comput. Electron. Agric..

[161] Nguyen TT, Kayacan E, Baedemaeker JD, Saeys W (2013). Task and motion planning for apple harvesting robot*. IFAC Proc..

[162] Hornung A, Wurm KM, Bennewitz M, Stachniss C, Burgard W (2013). Octomap: an efficient probabilistic 3d mapping framework based on octrees. Auton. Robot..

[163] Mehta SS, Burks TF (2014). Vision-based control of robotic manipulator for citrus harvesting. Comput. Electron. Agric..

[164] Barth R, Hemming J, van Henten EJ (2016). Design of an eye-in-hand sensing and servo control framework for harvesting robotics in dense vegetation. Biosyst. Eng..

[165] Ringdahl O, Kurtser P, Edan Y (2019). Evaluation of approach strategies for harvesting robots: Case study of sweet pepper harvesting: Category: (5). J. Intell. Robot. Syst..

[166] Ringdahl, O., Kurtser, P., Edan, Y.: Strategies for selecting best approach direction for a sweet-pepper harvesting robot. **10454**, 516–525. 10.1007/978-3-319-64107-2_41 (2017)

[167] Camacho, J.D.G., From, P.J., Leite, A.C.: A Visual Servoing Approach for Robotic Fruit Harvesting in the Presence of Parametric Uncertainties XXII Congresso Brasileiro De Automatica. 1. Campinas/SP Brasil: SBA. 10.20906/cps/cba2018-0541 (2018)

[168] Bu L, Hu G, Chen C, Sugirbay A, Chen J (2020). Experimental and simulation analysis of optimum picking patterns for robotic apple harvesting. Sci. Hortic..

[169] Wei, J., Yi, D., Bo, X., Guangyu, C., Dean, Z.: Adaptive variable parameter impedance control for apple harvesting robot compliant picking. Complexity, 1–15. 10.1155/2020/4812657 (2020)

[170] Roshanianfard A, Noguchi N (2018). Characterization of pumpkin for a harvesting robot. IFAC-PapersOnLine.

[171] Mehta SS, MacKunis W, Burks TF (2014). Nonlinear robust visual servo control for robotic citrus harvesting. IFAC Proceedings.

[172] Vasconez JP, Kantor GA, Cheein FAA (2019). Human–robot interaction in agriculture: a survey and current challenges. Biosyst. Eng..

[173] Bechar A, Vigneault C (2016). Agricultural robots for field operations: Concepts and components. Biosyst. Eng..

[174] Adamides, G.: Doctoral dissertation: user interfaces for human-robot interaction: Application on a semi-autonomous agricultural robot sprayer. Ph.D dissertation (2016)

[175] Bergerman M, Maeta SM, Zhang J, Freitas GM, Hamner B, Singh S, Kantor G (2015). Robot farmers: Autonomous orchard vehicles help tree fruit production. IEEE Robotics Automation Magazine.

[176] Cullen RH, Smarr C-A, Serrano-Baquero D, McBride SE, Beer JM, Rogers WA (2012). The smooth (tractor) operator: Insights of knowledge engineering. Appl. Ergon..

[177] Jin X, Zheng B, Pei Y, Li H (2017). A method to estimate operator’s mental workload in multiple information presentation environment of agricultural vehicles. Engineering Psychology and Cognitive Ergonomics: Performance Emotion and Situation Awareness.

[178] Gomez-Gil J, San-Jose-Gonzalez I, Nicolas-Alonso LF, Alonso-Garcia S (2011). Steering a tractor by means of an emg-based human-machine interface. Sensors.

[179] Szczepaniak J, Tanas W, Pawlowski T, Kromulski J (2014). Modelling of agricultural combination driver behaviour from the aspect of safety of movement. Annals of Agricultural and Environmental Medicine: AAEM.

[180] Mohan J, Lanka K, Rao NA (2019). A review of dynamic job shop scheduling techniques. Procedia Manuf..

[181] Petrovic, M., Miljkovic, Z., Jokic, A.: A novel methodology for optimal single mobile robot scheduling using whale optimization algorithm. Applied Soft Computing Journal, 81. 10.1016/j.asoc.2019.105520(2019)

[182] Turkyilmaz, A., Senvar, O., Unal, I., Bulkan, S.: A research survey: heuristic approaches for solving multi objective flexible job shop problems. Journal of Intelligent Manufacturing 31(4). 10.1007/s10845-020-01547-4 (2020)

[183] Xu, L., Jiawei, D., Ming, H.: Research on hybrid cloud particle swarm optimization for multi-objective flexible job shop scheduling problem (2017)

[184] Li JQ, Pan QK, Tasgetiren MF (2014). A discrete artificial bee colony algorithm for the multi-objective flexible job-shop scheduling problem with maintenance activities. Appl. Math. Model..

[185] Zheng Y, Li YX, Lei DM (2012). Multi-objective swarm based neighborhood search of fuzzy flexible job shop scheduling. The international Journal of Advanced Manufacturing Technologies.

[186] Li JQ, Duan P, Cao J, Li XP, Pan YY (2018). A hybrid pareto based tabu search for the distributed flexible job shop scheduling problem with e/t criteria. IEEE Access.

[187] Huang RH, Yang CL, Cheng WC (2013). Flexible job shop scheduling with due window- a two-pheromone ant colony approach. Int. J. Prod. Econ..

[188] Reddy MB, Ratnam C, Rajyalakshmi G, Manupati VK (2019). An effective hybrid multi objective evolutionary algorithm for solving real time event in flexible job shop scheduling problem. Measurement.

[189] Huang X, Yang L (2019). A hybrid genetic algorithm for multi objective flexible job shop scheduling problem considering transportation time. International Journal of Intelligent Computing and Cybernetics.

[190] Ojstersek R, Zhang H, Liu S, Buchmeister B (2018). Improved heuristic kalman algorithm for solving multi-objective flexible job-shop scheduling problem. Procedia Manufacturing.

[191] Zhou Y, Yang J, Zheng L (2019). Multi-agent based hyper-heuristics for multi-objective flexible job-shopscheduling: a case study in an aero-engine blade manufacturing plant. IEEE Access.

[192] Seyyedhasani, H., Peng, C., Jang, W. -J., Vougioukas, S.G.: Collaboration of human pickers and crop-transporting robots during harvesting – part i: Model and simulator development Computers and Electronics in Agriculture 172. 10.1016/j.compag.2020.105324 (2020)

[193] Conesa-Muñoz J, Bengochea-Guevara JM, Andujar D, Ribeiro A (2016). Route planning for agricultural tasks: a general approach for fleets of autonomous vehicles in site-specific herbicide applications. Comput Electron Agric..

[194] Edwards G, Sorensen CG, Bochtis D, Munkholm LJ (2015). Optimized schedules for sequential agricultural operations using a tabu search method. Comput. Electron. Agric..

[195] Ahsan Z, Dankowicz H (2019). Optimal scheduling and sequencing for large-scale seeding operations. Comput. Electron. Agric..

[196] Jensen MF, Bochtis D, Sorensen CG (2015). Coverage planning for capacitated field operations, part ii: Optimisation. Biosyst. Eng..

[197] Santoro E, Soler EM, Cherri AC (2017). Route optimization in mechanized sugarcane harvesting. Comput. Electron. Agric..

[198] Cheein FA, Torres-Torriti M, Hopfenblatt NB, Prado AJ, Calabi D (2017). Agricultural service unit motion planning under harvesting scheduling and terrain constraints. Journal of Field Robotics.

[199] Richards, D., Patten, T., Fitch, R.C., Ball, D., Sukkarieh, S.: User interface and coverage planner for agricultural robotics. Australasian Conference on Robotics and Automation (2015)

[200] Mann MP, Zion B, Shmulevich I, Rubinstein D, Linker R (2016). Combinatorial optimization and performance analysis of a multi-arm cartesian robotic fruit harvester–extensions of graph coloring. J. Intell. Robot. Syst..

[201] Barnett J, Duke M, Au CK, Lim SH (2020). Work distribution of multiple cartesian robot arms for kiwifruit harvesting. Comput. Electron. Agric..

[202] Kurtser, P., Edan, Y.: Planning the sequence of tasks for harvesting robots. Robot. Auton. Syst., 131. 10.1016/j.robot.2020.103591 (2020)

[203] Miller, A.T., Allen, P.K.: Examples of 3d grasp quality computations. **2**, 1240–1246. 10.1109/ROBOT.1999.772531 (1999)

[204] Rodríguez F, Moreno JC, Sánchez JA, Berenguel M (2012). Grasping in agriculture: State-of-the-art and main characteristics. Mechanisms and Machine Science.

[205] Mu L, Cui G, Liu Y, Cui Y, Fu L, Gejima Y (2020). Design and simulation of an integrated end-effector for picking kiwifruit by robot. Information Processing in Agriculture.

[206] Liu, J., Li, P., Li, Z.: A multi-sensory end-effector for spherical fruit harvesting robot, 258–262. 10.1109/ICAL.2007.4338567 (2007)

[207] Jia, B., Zhu, A., Yang, S.X., Mittal, G.S.: Integrated gripper and cutter in a mobile robotic system for harvesting greenhouse products, 1778–1783. 10.1109/ROBIO.2009.5420430 (2009)

[208] Dimeas F, Sako DV, Moulianitis V, Aspragathos N (2014). Design and fuzzy control of a robotic gripper for efficient strawberry harvesting. Robotica.

[209] Zhong H, Nof SY, Berman S (2015). Asynchronous cooperation requirement planning with reconfigurable end-effectors. Robotics and Computer Integrated Manufacturing.

[210] Pedrazzoli, P., Rinaldi, R., Boer, C.R.: A rule based approach to the gripper selection issue for the assembly process. 202–207. 10.1109/ISATP.2001.928990 (2001)

[211] Pham DT, Gourashi NS, Eldukhri EE (2007). Automated configuration of gripper systems for assembly tasks. Proc. Ins. Mech. Eng. Part B: J. Eng. Manuf..

[212] Sanfilippo, F., Salvietti, G., Zhang, H.X., Hildre, H.P., Prattichizzo, D.: Efficient modular grasping: An iterative approach, 1281–1286. 10.1109/BioRob.2012.6290693 (2012)

[213] Brown, R.G., Brost, R.C.: A 3d modular gripper design tool. **3**, 2332–2339. 10.1109/ROBOT.1997.619310 (1997)

[214] Balan L, Bone GM (2003). Automated gripper jaw design and grasp planning for sets of 3d objects. Journal of Field Robotics.

[215] Velasco, V.B., Newman, W.S.: An approach to automated gripper customization using rapid prototyping technology (1996)

[216] Velasco, V.B., Newman, W.S.: Computer-assisted gripper and fixture customization using rapid-prototyping technology, **4**, 3658–3664. 10.1109/ROBOT.1998.681393 (1998)

[217] Honarpardaz, M., Tarkian, M., Feng, X., Sirkett, D., Ölvander, J.: Generic automated finger design. **5**,1–9. 10.1115/DETC2016-60514 (2016)

[218] Sahbani A, El-Khoury S, Bidaud P (2012). An overview of 3d object grasp synthesis algorithms. Robot. Auton. Syst..

[219] Bicchi, A., Kumar, V.: Robotic grasping and contact: a review. **1**, 348–353. 10.1109/ROBOT.2000.844081 (2000)

[220] Li J-W, Liu H, Cai H-G (2003). On computing three-finger force-closure grasps of 2-d and 3-d objects. IEEE Trans. Robot. Autom..

[221] Han, L., Trinkle, J.C., Li, Z.: Grasp analysis as linear matrix inequality problems. 1261–1268. 10.1109/ROBOT.1999.772534 (1999)

[222] Mishra B, Schwartz JT, Sharir M (1987). On the existence and synthesis of multifinger positive grips. Algorithmica.

[223] Liu Y-H (1999). Qualitative test and force optimization of 3-d frictional form-closure grasps using linear programming. IEEE Trans. Robot. Autom..

[224] Borst, C., Fischer, M., Hirzinger, G.: Grasping the dice by dicing the grasp. **3**, 3692–3697. 10.1109/IROS.2003.1249729 (2003)

[225] Miller, A.T., Knoop, S., Christensen, H.I., Allen, P.K.: Automatic grasp planning using shape primitives. 2:1824–1829. 10.1109/ROBOT.2003.1241860 (2003)

[226] Ding, D., Liu, Y.-H., Wang, S.: Computing 3-d optimal form-closure grasps. **4**, 3573–3578. 10.1109/ROBOT.2000.845288 (2000)

[227] Ding, D., Liu, Y.-H., Wang, M.Y.: On computing immobilizing grasps of 3-d curved objects. In: Proceedings 2001 IEEE International Symposium on Computational Intelligence in Robotics and Automation, pp. 11–16. 10.1109/CIRA.2001.1013165 (2001)

[228] Liu Y-H, Lam M-L, Ding D (2004). A complete and efficient algorithm for searching 3-d form-closure grasps in the discrete domain. IEEE Trans. Robot..

[229] Eizicovits D, Berman S (2014). Efficient sensory-grounded grasp pose quality mapping for gripper design and online grasp planning. Robot. Auton. Syst..

[230] Liu, S., Carpin, S.: Global grasp planning using triangular meshes, 4904–4910. 10.1109/ICRA.2015.7139880 (2015)

[231] Hemming, J., Bac, C.W., Tuijl, B., Barth, R., Bontsema, J., Pekkeriet, E., Henten, E.V.: A robot for harvesting sweet-pepper in greenhouses (2014)

[232] Bohg J, Morales A, Asfour T, Kragic D (2014). Data-driven grasp synthesis - a survey. IEEE Trans. Robot..

[233] Kim J, Iwamoto K, Kuffner JJ, Ota Y, Pollard NS (2013). Physically based grasp quality evaluation under pose uncertainty. IEEE Trans. Rob..

[234] Wolniakowski, A., Miatliuk, K., Kruger, N., Rytz, J.A.: Automatic evaluation of task-focused parallel jaw gripper design. International Conference on Simulation, Modeling, and Programming for Autonomous Robots, 450–461. 10.1007/978-3-319-11900-7_38 (2014)

[235] Fernández R, Salinas C, Montes H, Sarria J (2014). Multisensory system for fruit harvesting robots. experimental testing in natural scenarios and with different kinds of crops. Sensors.

[236] Bac CW, Hemming J, van Tuijl BAJ, Barth R, Wais E, van Henten EJ (2017). Performance evaluation of a harvesting robot for sweet pepper. Journal of Field Robotics.

[237] Tardáguila, J., Blasco, J., Diago, M.P.: Vinerobot: A new robot for vineyard monitoring using non-invasive sensing technologies. In: 9th International Cool Climate Wine Symposium. Retrieved from https://digital.csic.es/handle/10261/148399 - Last accessed: 19-05-2022 (2016)

[238] Fernández-Novales J, Garde-Cerdán T, Tardáguila J, Gutiérrez-Gamboa G, Pérez-Álvarez EP, Diago MP (2019). Assessment of amino acids and total soluble solids in intact grape berries using contactless vis and nir spectroscopy during ripening. Talanta.

[239] Aquino A, Millan B, Diago MP, Tardaguila J (2018). Automated early yield prediction in vineyards from on-the-go image acquisition. Comput. Electron. Agric..

[240] Fernández-Novales J, Tardaguila J, Gutiérrez S, Marañón M, Diago MP (2018). In field quantification and discrimination of different vineyard water regimes by on-the-go nir spectroscopy. Biosyst. Eng..

[241] Lopes, C.M., Graça, J.D., Sastre, J., Reyes, M., Guzman, R., Braga, R., Monteiro, A., Pinto, P.A.: Vineyard yield estimation by vinbot robot - preliminary results with the white variety viosinho. 10.13140/RG.2.1.3912.0886 (2016)

[242] Lopes, C.M., Torres, A., Guzman, R., Graça, J. D., Monteiro, A., Braga, R.P., Barriguinha, A., Victorino, G., Reys, M.: Using an unmanned ground vehicle to scout vineyards for non-intrusive estimation of canopy features and grape yield. 20th giESCO International Meeting (2017)

[243] Astolfi P, Gabrielli A, Bascetta L, Matteucci M (2018). Vineyard autonomous navigation in the echord++ grape experiment. IFAC-PapersOnLine.

[244] Leu A, Razavi M, Langstädtler L, Ristić-Durrant D, Raffel H, Schenck C, Gräser A, Kuhfuss B (2017). Robotic green asparagus selective harvesting. IEEE/ASME Trans. Mechatron..

[245] Fernández-Novales J, Tardáguila J, Gutiérrez S, Diago MP (2019). On-the-go vis + sw − nir spectroscopy as a reliable monitoring tool for grape composition within the vineyard. Molecules.

[246] Gutiérrez S, Tardaguila J, Fernández-Novales J, Diago MP (2019). On-the-go hyperspectral imaging for the in-field estimation of grape berry soluble solids and anthocyanin concentration. Aust. J. Grape Wine Res..

[247] Gutiérrez S, Diago MP, Fernández-Novales J, Tardaguila J (2017). On-the-go thermal imaging for water status assessment in commercial vineyards. Adv. Anim. Biosci..

[248] Grimstad, L., Pham, C.D., Phan, H.T., From, P.J.: On the design of a low-cost, light-weight, and highly versatile agricultural robot. IEEE Workshop on Advanced Robotics and its Social Impacts ARSO. 10.1109/ARSO.2015.7428210 (2016)

[249] Harvest Croo Robotics. https://harvestcroo.com/ - Last accessed: 22-11-2022

[250] Dogtooth. https://dogtooth.tech/ - Last accessed: 22-11-2022

[251] Agrobot E-Series. http://agrobot.com - Last Accessed: 22-11-2022

[252] OCTINION. http://octinion.com/products/agricultural-robotics/rubion - Last Accessed: 22-11-2022

[253] SAGA Robotics. https://sagarobotics.com - Last Accessed: 22-11-2022

[254] Grimstad, L., From, P.J.: The thorvald ii agricultural robotic system. Robotics 6(4). 10.3390/robotics6040024 (2017)

[255] MetoMotion. https://metomotion.com- Last accessed: 22-11-2022

[256] Root-AI. https://www.appharvest.com/press_release/appharvest-acquires-agricultural-robotics-and-artificial-intelligence-company-root-ai-to-increase-efficiency/ - Last accessed: 22-11-2022

[257] AppHarvest. https://www.appharvest.com/ - Last accessed: 22-11-2022

[258] ENERGID. https://www.energid.com/industries/agricultural-robotics - Last accessed: 22-11-2022

[259] VISION ROBOTICS. https://www.visionrobotics.com/vr-grapevine-pruner - Last accessed: 22-11-2022

[260] naio Technologies. https://www.naio-technologies.com/en/ted/ - Last accessed: 22-11-2022

[261] ViTiBOT. https://vitibot.fr/en - Last Accessed: 22-11-2022

